# Leiomyosarcoma of the Penis

**DOI:** 10.1080/1357714021000022177

**Published:** 2002-06

**Authors:** Vasilios S. Katsikas, Konstantinos D. Kalyvas, Spiros S. Ioannidis, Michalis V. Papathanasiou, Konstantina P. Panagiotopoulou, Panagiotis M. Hitiroglou, Konstantinos Yannakoyorgos

**Affiliations:** 1 Department of Urology Medical School Aristoteles University of Thessaloniki Thessaloniki Greece; 2 Department of Pathology Medical School Aristoteles University of Thessaloniki Thessaloniki Greece; 3 27 Koritsas St. Thessaloniki GR 55535 Greece

## Abstract

We report a case of a 78-year-old patient with penile leiomyosarcoma, treated by radical penectomy. Two years after the operation the patient is without evidence of local recurrence or metastatic disease.We also discuss the treatment options and attempt a review of the literature.


*Sarcoma* (2002) **6**, 75–77

Taylor&Francis healthsciences

**CASE REPORT**

### Leiomyosarcoma of the penis

VASILIOS S. KATSIKAS[1], KONSTANTINOS D. KALYVAS[1], SPIROS S. IOANNIDIS[1], MICHALIS V. PAPATHANASIOU[1], KONSTANTINA P. PANAGIOTOPOULOU[2], PANAGIOTIS M. HITIROGLOU[2] & KONSTANTINOS YANNAKOYORGOS[1]

*Departments of [1]Urology and [2]Pathology, Medical School, Aristoteles University of Thessaloniki, Greece*

**Abstract**

We report a case of a 78-year-old patient with penile leiomyosarcoma, treated by radical penectomy. Two years after the operation the patient is without evidence of local recurrence or metastatic disease. We also discuss the treatment options and attempt a review of the literature.

**Key words:** *leiomyosarcoma, penis, deep, surgery*

### Introduction

Leiomyosarcoma is a rare tumor of the penile mesechymal tissue. Because of the small number of cases reported so far, the conclusions about treatment and prognosis are equivocal.[1] We report an additional case, and attempt a review in the literature.

### *Case report*

A 78-year-old farm worker presented with a 3-year history of gradual painless swelling of the penis. His medical history was remarkable for generalized vascular disease and diabetes mellitus. During the physical examination a non-mobile hard mass was palpated involving the base and the midshaft of the penis. The glans penis was normal and no regional lymphadenopathy was found. Serum biochemistry and full blood count were normal. A CT showed a soft mass lesion involving the corpora cavernosa of the penis, but no evidence of metastatic disease. With the presumptive diagnosis of penile sarcoma the patient underwent a radical penectomy with perineal urethrostomy. Macroscopically, a tumor measuring $8 \times 8 \times 14$ cm was found to arise from the corpora, making the distinction between them almost impossible. The urethra and glans were free of invasion (Fig. 1). Microscopically, the tumor had the features of high-grade sarcoma, being composed of neoplastic spindle-shaped cells, with eosinophilic cytoplasm arranged in fascicles. The cells showed moderate nuclear atypia, with hyperchromatic nuclei and the mitotic rate was five per high-power field. On immunohistochemical staining, the tumor cells were positive for vimentin and SMA (smooth muscle antigen) (Fig. 2) and negative for desmin and S-100 protein. In conclusion, the tumor proved to be a leiomyosarcoma. The patient made an uneventful recovery and is well, with no evidence of disease, 2 years after the operation.

### Discussion

The most common primary malignant neoplasm of the penis is squamous cell carcinoma, followed by metastatic neoplasms such as prostate, bladder, rectum, kidney and testis, and those spreading by direct extension from the adjacent structures. Mesenchymal neoplasms are rare and represent less than 5% of all types of penile malignant disease.[2]

According to Dehner and Smith, who in their classic review[3] analyzed 46 primary soft tissue tumors of the penis, leiomyosarcoma represents approximately 13.5 and 6.5% of penile sarcomas and soft tissue tumors in general, respectively. This is in concordance with the earlier results of Ashley and Edwards[4] where they found an incidence of 5.5%.

To the best of our knowledge, the last well-documented case was that of Pow-Sang and Orihuela in 1994[5] and, according to their review, the present case is the 20th of the deep-seated lesions and the 28th that has been reported in general.

The age range at diagnosis is from 6 years[6] to the late 80s.[7]

Correspondence to: Dr. Konstantinos Kalyvas, 27 Koritsas St., GR 55535 Thessaloniki, Greece. E-mail: kalivas@spark.net.gr

ISSN 1357–714X print/ISSN 1369–1643 online/02/0200075–03 © Taylor & Francis Ltd
DOI: 10.1080/1357714021000022177

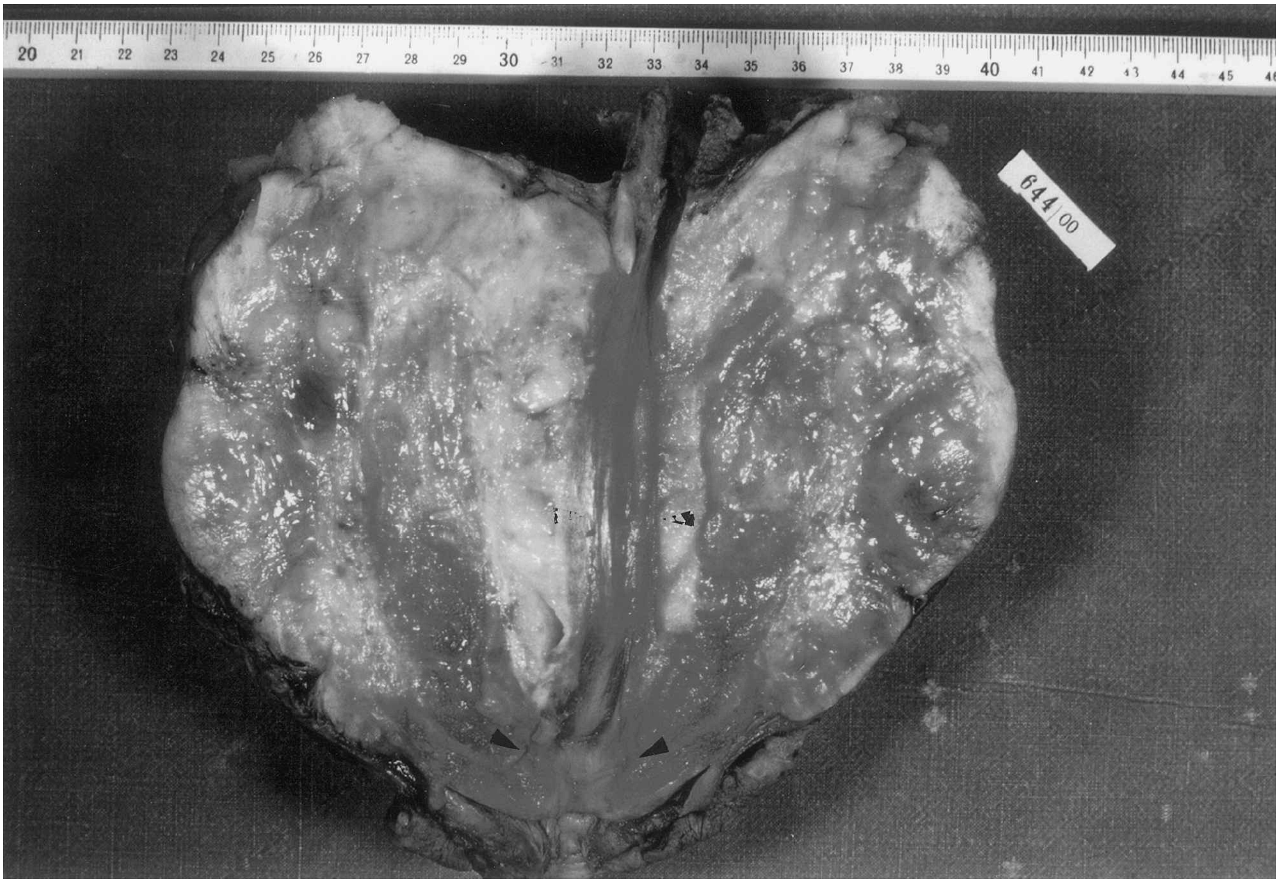

**Fig. 1.** *Macroscopic view of the excised tumor, arrowheads indicating the glans penis at the bottom. Note that the urethra is free of invasion. The tumor size is estimated to be approximately 18 cm.*

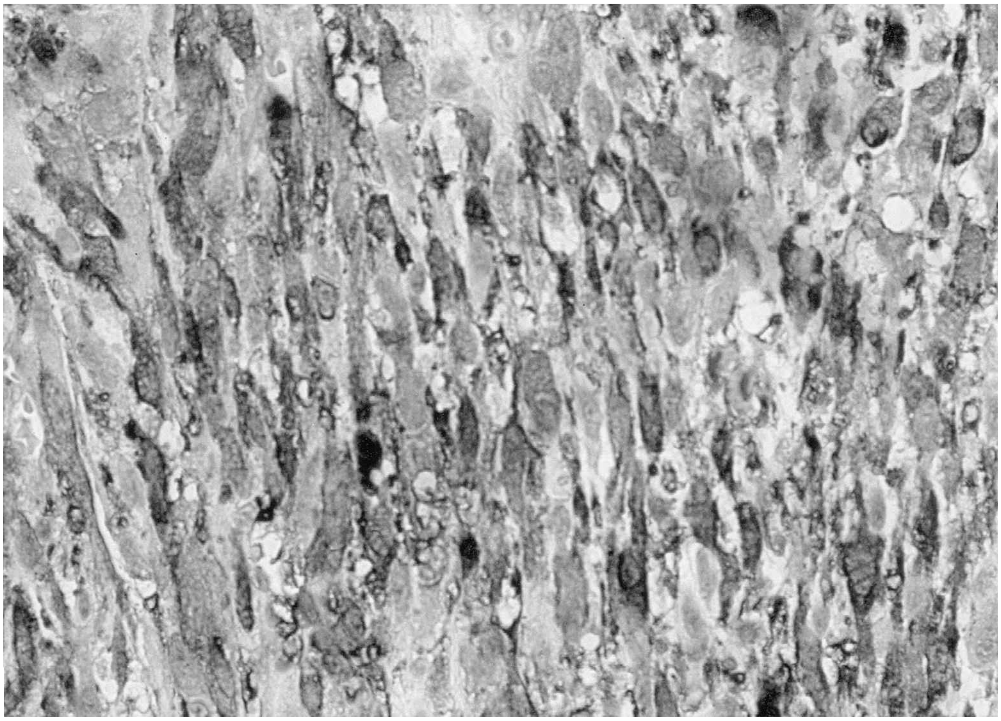

**Fig. 2.** *On immunohistochemical stains, strong positivity for smooth muscle antigen (SMA, 100) can be seen.*

Pratt and Ross[7] were the first authors who classified penile leiomyosarcomas into two distinct pathological and clinical entities, superficial and deep-seated tumors.

Superficial lesions usually present as a tumorlet in the distal shaft or the penile prepuce, often in middle-aged men, and commonly it is a slowly growing tumor, with low metastatic potential.

Deep lesions, one of which we present here, arise from the glans penis and from the proximal portions of the corpora cavernosa or corpus spongiosum, and occur at a relatively more advanced age.

In contrast to the superficial tumors, the deep lesions show greater propensity to metastasize and have therefore poorer prognosis. Clinically they are poorly circumscribed, firm non-tender masses that infiltrate surrounding tissues and can be the cause of urinary obstruction or urethrocutaneous fistula.[8]

The origin of the superficial type neoplasm is presumed to be the erector pilorum muscle of the dermis or the smooth muscular elements of the subcutaneous tissue. The deep-seated lesions possibly arise from the smooth muscle cells of the corpora cavernosa or the corpus spongiosum, or they may be due to progression of an initially superficial lesion.[5] For the tumors arising in the glans penis the origin could be from blood vessel walls.[6]

On gross section they are usually rubbery in consistency, well circumscribed and with white, yellow, or gray appearance.

Microscopically, they are composed of spindle-shaped smooth muscle fibers arranged into interlacing fasicles. The importance of mitotic rate and other nuclear differentiation variables, have not been analyzed in the literature, but the data show that the degree of differentiation is reliable in order to predict the tumor propensity to infiltrate the adjacent structures or to metastasize. Histologically the two types are identical.

On electron microscopy examination myofibrils, dense bodies, and abundant pinocytic vesicles are noted, and a continuous basal lamina is present around most of the tumor cells.[9]

It seems that, of mesenchymal penile tumors, leiomyosarcomas are more prone to recur and they become more undifferentiated with each recurrence,[1] The recurrence rate is relatively similar in both groups, but the metastatic potential is higher in deep-seated lesion.[3]

The treatments of choice are (1) surgery, in the form of local excision, amputation whether partial or total, or radical penectomy, (2) radiation, or (3) chemotherapy. Surgery should aim at the excision of the tumor mass. Amputation, is the most effective treatment to prevent recurrences for both types of penile leiomyosarcoma,[5] but the approach should be individualized, and because superficial tumors tend to appear in younger men, these cases can be managed by local excision with negative surgical margins whenever this is possible. Deep lesions are most appropriately treated with a more aggressive approach, namely amputation for the distal lesions, or radical penectomy for the middle or proximal shaft lesions.

In contrast to squamous cell carcinoma of the penis where the excision of the regional lymph nodes can be considered curative in early stages, this is not the case in penile leiomyosarcoma,[10] and the radiation or excision of regional lymph nodes cannot be considered to have an influence on patient survival.[5]

Pre- or postoperative external beam radiation has not proved its value in treating penile leiomyosarcomas or in increasing survival rates.[11] Brachytherapy has not yet been reported as a treatment. Chemotherapy with anthracyclines or etoposide has provided poor results and, unfortunately, ongoing trials show that the newer taxanes, have not been successful in treating uterine leiomyosarcomas.[12]

Nevertheless, both treatment modalities can be used for palliation in recurrences not amenable to surgical treatment.

In conclusion, leiomyosarcoma of the penis is a very rare disease cured mainly by surgical intervention. The effectiveness of radiation therapy and chemotherapy is debatable, and the lack of large series makes the conclusions insecure. Because of the small number of cases so far, the best approach for these malignant tumors is the collaboration between the urological surgeon, the pathologist, the radiotherapist and the medical oncologist in order to optimize the results for the best interest of the patient.

### References

1. Valadez RA, Waters WB. Leiomyosarcoma of penis. *Urology* 1986; 27(3): 265–7.
2. Lucia MS, Miller GJ. Histopathology of the malignant lesions of the penis. *Urol Clin N Am* 1992; 19: 227.
3. Dehner LP,Smith BH. Soft tissue tumors of the penis. A clinicopathologic study of 46 cases. *Cancer* 1970; 25: 1431–47.
4. Ashley DJ, Edwards EC. Sarcoma of the penis. Leiomyosarcoma of the penis. *Br J Surg* 1957; 45: 170.
5. Pow-Sang MR, Orihuela E . Leiomyosarcoma of the penis. *J Urol* 1994; 151(6): 1643–5.
6. Glucker E, Hirshowitz B, Gellei B. Leiomyosarcoma of the glans penis. Case report. *Plast Reconstr Surg* 1972; 50(4): 406–8.
7. Pratt RM, Ross RT. Leiomyosarcoma of the penis. A report of a case. *Br J Surg* 1969; 56(11): 870–2.
8. Nkposong EO, Osunkoya BO. Leiomyosarcoma of the penis. *West Africa Med J* 1972; 21: 32.
9. Kathuria S, Jablokow VR, Molnar Z. Leiomyosarcoma of the penile prepuce with ultrastructural study. *Urology* 1986; 27(6): 556–7.
10. SrivinasV, Morse MJ, Herr HW, Sogani PC, Whitmore WF Jr. Penile cancer: relation of extent of nodal metastasis to survival. *J Urol* 1987; 137: 880.
11. Greenwood N, Fox H, Edwards EC. Leiomyosarcoma of the penis. *Cancer* 1972; 29(2): 481–3.
12. Sutton G, Blessing JA, Ball H. Phase II trial of paclitaxel in leiomyosarcoma of the uterus: a gynecological oncology group study. *Gynecol Oncol* 1999; 74(3): 346–9.
